# The Assessment of Body Image Based on Large Language Model

**DOI:** 10.1002/pchj.70048

**Published:** 2025-08-30

**Authors:** Fumeng Li, Nan Zhao

**Affiliations:** ^1^ State Key Laboratory of Cognitive Science and Mental Health, Institute of Psychology, Chinese Academy of Sciences Beijing China; ^2^ Office of International Affairs, Tsinghua University Beijing China

**Keywords:** body image, criterion validity, ecological validity, large language models, prompt engineering

## Abstract

Assessing adolescent body image is crucial for mental health interventions, yet traditional methods suffer from limited dimensional coverage, poor dynamic tracking, and weak ecological validity. To address these gaps, this study proposes a multidimensional evaluation using large language models (LLMs) and compares its criterion validity against a dictionary‐based method and expert ratings. We defined four dimensions—perception, positive attitude, negative attitude, behavior—by reviewing the body‐image literature and built a validated dictionary through expert ratings and iterative refinement. A four‐step prompt‐engineering process, incorporating role‐playing and other optimization techniques, produced tailored prompts for LLM‐based recognition. To validate these tools, we collected self‐reported texts and scale scores from 194 university students, performed semantic analyses with Llama‐3.1‐70B, Qwen‐Max, and DeepSeek‐R1 using these prompts, and confirmed ecological validity on social media posts. Results indicate that our multidimensional dictionary correlated significantly with expert ratings across all four dimensions (*r* = 0.515–0.625), providing a solid benchmark. LLM‐based assessments then outperformed both the dictionary and human ratings, with zero‐shot LLMs achieving *r* = 0.664 in positive attitude (vs. expert *r* = 0.657) and DeepSeek‐R1 reaching *r* = 0.722 in perception. Role‐playing techniques significantly improved the validity in the perception dimension (Δ*r* = +0.117). Consistency checks revealed that the DeepSeek model reduced error dispersion in extreme score ranges by 48.4% compared to human ratings, with the 95% consistency limits covering the fluctuations of human scores. Incremental validity analysis showed that LLMs could replace human evaluations in the perception dimension (Δ*R* ^2^ = 0.220). In ecological validity checks, the Qwen model achieved a correlation of 0.651 in the social media behavior dimension—53.1% higher than the dictionary method. We found that LLMs demonstrated significant advantages in the multidimensional assessment of body image, offering a new intelligent approach to mental health measurement.

## 1 Introduction

Body image refers to an individual's perception, attitudes, emotions, and behavioral tendencies related to their body. It encompasses multiple dimensions, including body appearance, body functionality, emotional experiences related to the body, and behavioral responses elicited by bodily perceptions (Cash and Fleming 2002; Schilder 1950). It is a crucial component of psychological health, closely associated with self‐esteem, emotions, interpersonal relationships, and overall well‐being (Thompson and Smolak 2001). Negative body image has been found to contribute to body shame (Fredrickson and Roberts 1997), heightened anxiety (Zheng et al. 2015), reduced psychological well‐being (Frison and Eggermont 2016), and even depression (Zhao 2020). Moreover, body dissatisfaction has been shown to have a strong association with eating disorders (OR = 4.2), social anxiety (*β* = 0.470), and suicidal ideation (RR = 2.1) (Bornioli et al. 2021).

Adolescence is marked by rapid endocrine and somatic changes alongside a developing “social brain,” leading to heightened self‐consciousness, social comparison, and sensitivity to peer evaluation and societal ideals of thinness (Jones and Smolak 2011). Emerging adulthood (ages 18–25), the life stage of most university students, is distinguished by identity exploration, instability, self‐focus, feeling “in‐between,” and a sense of possibilities. In the university context, these features intersect with newfound autonomy, shifting peer networks, and lifestyle transitions—such as meal pattern changes and novel social environments—that together intensify body image concerns (Huguenin et al. 2024). Consequently, young adults in higher education face unique vulnerabilities as they negotiate self‐definition and societal appearance norms (Gillen 2007). Such concerns can lead to various psychological issues, including depression, anxiety, and eating disorders (Levine and Smolak 2006), which in turn can negatively impact their academic performance, social integration, and interpersonal relationships. Epidemiological data indicate that the prevalence of body dissatisfaction among individuals aged 13–25 ranges from 38% to 60%, with significant correlations to anxiety, depression, and eating disorders (Fiske et al. 2014). Therefore, the accurate assessment of body image is crucial for promoting adolescents' psychological well‐being.

Traditional methods for assessing body image include self‐report scales, image‐based methods, and observational techniques. Commonly used questionnaires, such as the Multidimensional Body‐Self Relations Questionnaire (MBSRQ, Brown et al. 1990; Cash and Fleming 2002) and the Body Appreciation Scale (BAS‐2, Tylka and Wood‐Barcalow 2015a) have repeatedly demonstrated high internal consistency, test‐retest reliability, and construct validity across adolescent and adult samples. However, in large‐scale studies or situations requiring rapid assessment, these traditional methods are time‐consuming and resource‐intensive, making them unsuitable for handling large samples or time‐sensitive scenarios. Moreover, they are limited in capturing the dynamic fluctuations in body image over time (Cash and Fleming 2002). The multidimensional nature of body image has resulted in a high degree of diversity in measurement tools, with over 150 distinct instruments currently available (Plimpton and Thompson 2012). Existing methods tend to assess isolated dimensions, with an overemphasis on evaluative components (e.g., BAS). Consequently, more than 80% of these 150 instruments primarily focus on body satisfaction, while neglecting aspects such as behavioral engagement and perceptual specificity (Cash and Smolak 2011). Furthermore, most tools, such as the Body Image Affect Scale, primarily assess negative emotions, with only 15% incorporating positive constructs. This imbalance has contributed to a research lag in the study of positive body image (Tylka and Wood‐Barcalow 2015b).

Moreover, with the widespread adoption of social media among adolescents, traditional dimensions of body image struggle to account for emerging body‐related concerns. For instance, the prevalence of muscle dysmorphia among male adolescents has reached 17% (Martenstyn et al. 2023); however, existing measurement scales lack corresponding items to assess this condition. To address these limitations, previous research has developed text analysis tools, such as body image‐related dictionaries, to quantify body image concerns embedded in social media data (Ji et al. 2024). However, dictionary‐based approaches rely on simple word frequency statistics and are unable to interpret complex linguistic structures, such as irony or metaphor (Tiggemann et al. 2020). Additionally, these methods fail to fully capture subtle and nuanced expressions present in individuals' natural language. Furthermore, dictionary‐based tools struggle to update their corpora in a timely manner, making them inadequate for understanding new and evolving semantic expressions emerging on social media.

In recent years, artificial intelligence (AI), particularly large language models (LLMs), has opened new avenues for analyzing psychological characteristics in textual content. For instance, Wang, Li, et al. (2023) found that LLMs demonstrated emotional intelligence (EQ) scores exceeding the human average, suggesting their potential to detect subtle psychological states within text. Furthermore, Xu et al. (2024) demonstrated that models such as GPT‐4 exhibit significant potential in psychological reasoning tasks, indicating that advanced language models can comprehend and analyze complex psychological structures (Xu et al. 2024).

In the domain of mental health detection, Transformer‐based models, which leverage neural network architectures integrating metadata and linguistic markers, have demonstrated notable success in identifying depression from social media posts. These models have achieved a substantial improvement in the weighted F1 score, reaching 84.15% (Kerasiotis et al. 2024). In emotion recognition tasks, an instruction‐tuned LLaMA model, incorporating an emotion‐specific encoder to integrate multi‐modal inputs, achieved an F1 score of 0.9036 in the MER2023‐SEMI challenge, significantly outperforming traditional lexicon‐based methods (Cheng et al. 2024). Regarding personal life satisfaction prediction, approaches combining LLMs with machine learning techniques demonstrated a stronger correlation with self‐reported Satisfaction With Life Scale (SWLS) scores (*r* = 0.542) compared to using LLMs alone (*r* = 0.491) or expert ratings (*r* = 0.455) (Huang et al. 2024). These findings further validate the potential of LLMs in extracting psycholinguistic features, reinforcing their applicability in psychological research.

Notably, role‐playing prompt techniques, which simulate expert perspectives (e.g., “as a clinical psychologist”), enhance LLMs' sensitivity to complex emotional cues (Wang, Peng, et al. 2023). Meanwhile, chain‐of‐thought techniques improve the logical consistency of evaluations by guiding the model through step‐by‐step reasoning (Xia et al. 2024). Thus, the deep semantic understanding capabilities of LLMs not only enable the identification of explicit keywords but also facilitate the interpretation of metaphors and emotional contradictions. These advancements offer promising pathways for body image assessment.

Building on this, the current study innovatively explores the application potential of LLMs in body image assessment. By systematically comparing prompts to capture the four core dimensions of body image, the study predicts individuals' body image based on self‐statements and performs a comparative analysis of the predictive efficacy of dictionary‐based methods, expert ratings, and LLM‐based assessments. The study aims to address three key questions:

*Do LLMs demonstrate superior criterion‐related validity compared to dictionary‐based methods and human ratings in multidimensional body image assessment?*

*How do prompt engineering strategies and model architectures differentially influence LLMs' predictive accuracy across body image dimensions?*

*Can LLM‐based evaluations maintain consistent ecological validity across structured self‐narratives and unstructured social media contexts?*

Finally, this study aims to develop and validate an intelligent, text‐based framework for multidimensional body image assessment by integrating LLMs with established psychometric benchmarks. By constructing a comprehensive body image dictionary, optimizing prompt‐engineering strategies, and systematically comparing LLM‐generated scores against expert ratings and questionnaire outcomes, we seek to (1) establish the feasibility of automated, scalable assessments; (2) advance theoretical understanding of linguistic markers underlying body image cognition; and (3) demonstrate practical utility for large‐scale screening and real‐world monitoring in clinical and educational settings. The significance of this work lies in its potential to overcome the logistical and ecological limitations of traditional methods, offering a rapid, adaptable tool that can dynamically track body image concerns in both structured self‐reports and unstructured social media environments.

## 2 Method

### 2.1 Participants

Two hundred six participants were recruited from a university student cohort via online recruitment. After detecting deceptive responses through lie detection questions and self‐reported texts, and after data cleaning, 194 valid samples were retained. After obtaining fully informed consent, participants completed a questionnaire that included demographic information and body image assessment scales, and were asked to write a self‐statement of at least 300 words. This statement described their views and feelings regarding their own body image, covering the four dimensions of perception, positive attitude, negative attitude, and behavior, while not including names, contact details, or any information that could compromise their anonymity. All data were anonymized prior to analysis to ensure participant privacy and data security, and the research protocol was reviewed and approved by the ethics committee of the Institute of Psychology, Chinese Academy of Sciences.

Data from a total of 194 participants were collected, with 62.4% being female. The average age of participants was 22.88 years (SD = 4.40). A summary of the demographic characteristics and descriptive statistics is presented in Table 1. The sample primarily consisted of young adults, and the average word count of participants' self‐reported texts exceeded 389 words, providing a substantial amount of material for assessing their body image.

**TABLE 1:** Descriptive statistics.

Variable	Category	*N*	Percentage (%)	Mean (SD)
Gender	Female	135	69.6%	
	Male	59	30.4%	
Education Level	High School	3	1.5%	
	Undergraduate	144	74.3	
	Graduate and above	47	24.2%	
Age				23.12 (4.90)
Self‐statement Word Count				389.64 (127.61)

### 2.2 Study Design

**FIGURE 1:**
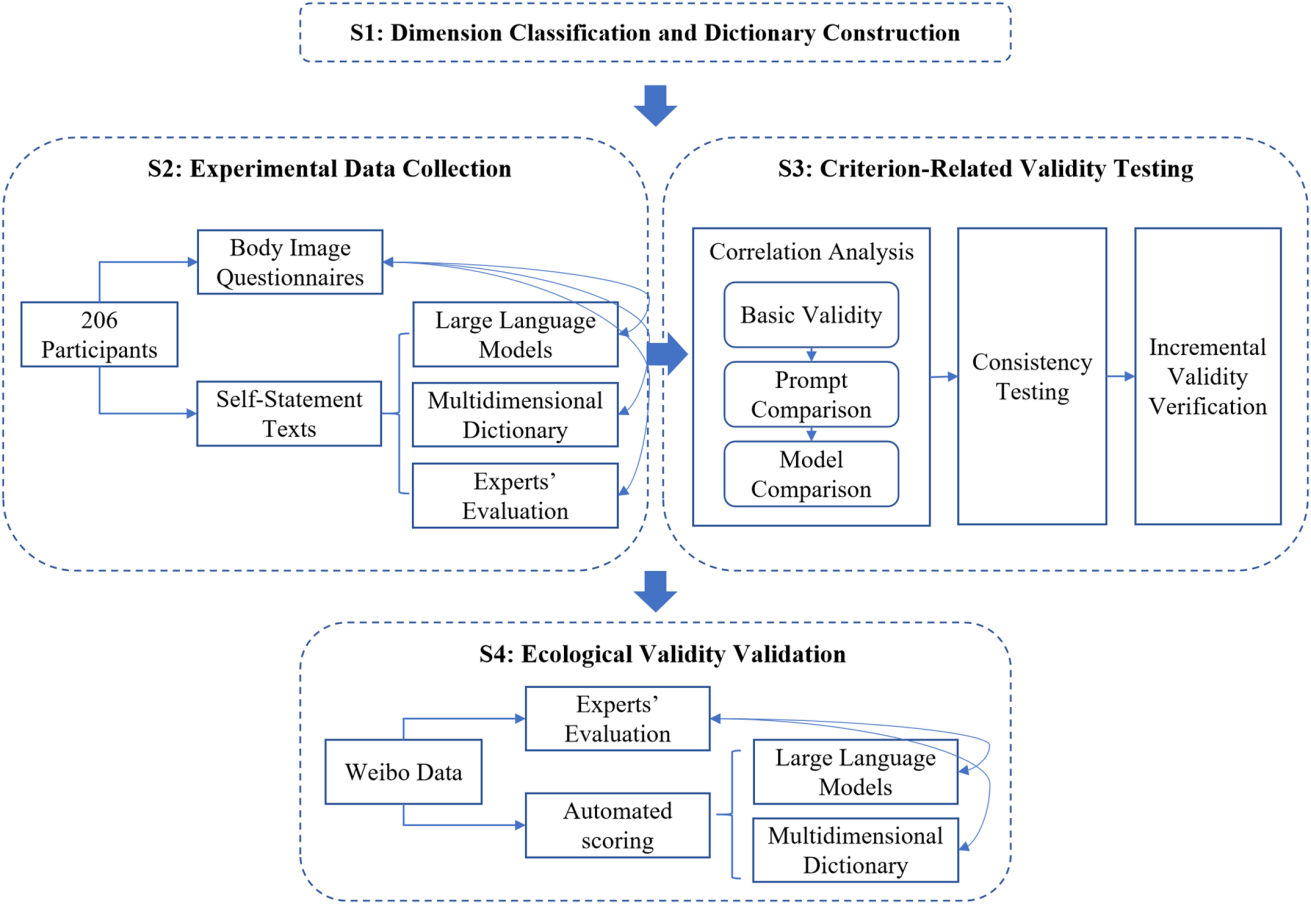
Overview of the research process.

This study was conducted in four stages: Multidimensional Scoring Tool Construction—Experimental Data Collection—Criterion‐Related Validity Testing—Ecological Generalization Test, as illustrated in Figure 1.

1. Stage 1: Dimension Classification and Dictionary Construction

Based on the framework of body image theory, four core dimensions—perception, positive attitude, negative attitude, and behavior—were established. A standardized dictionary was constructed as the assessment benchmark through literature integration and expert validation.

2 Stage 2: Experimental Data Collection

Participants' self‐statements and questionnaire data (*n* = 194) were collected as described above.

3 Stage 3: Criterion‐Related Validity Testing

LLMs were employed to assess participants' self‐statement texts and predict their scores across the body image dimensions. Additionally, two psychology experts manually scored the self‐statement texts. The analysis consisted of Correlation Analysis (basic validity, prompt engineering optimization, and model comparison), Consistency Testing, and Incremental Validity Verification.

4 Stage 4: Ecological Validity Validation

The generalization ability of the LLMs was tested in unstructured contexts by analyzing social media texts (Weibo).

### 2.3 Materials and Tools

The following subsections outline each source of data and its collection method—standardized body‐image scales, participant self‐statements, expert manual ratings, unstructured social media texts (Weibo), and LLM‐based scoring protocols.

1. *Body Image‐related Scales*: Based on the definition of body image, the cognitive, emotional (positive and negative), and behavioral aspects of body image were divided into four dimensions. For each dimension, questionnaire items capable of measuring the respective dimension were selected from the following sources: the “Appearance Evaluation,” “Appearance Orientation,” and “Overweight” sections of the Multidimensional Body‐Self Relations Questionnaire (MBSRQ); the Body Appreciation Scale (BAS‐2); and the Body Image Comprehensive Evaluation Questionnaire, which includes “Body Image Self‐Esteem,” “Body Image Concern,” “Body Image Emotion,” and “Body Image Behavior” sections. These items were used to obtain scale scores for each dimension of body image (see Table 2). The questionnaire used a Likert 5‐point scale (1 = *strongly disagree*, 5 = *strongly agree*). The scores for each dimension were calculated as the average of the relevant item scores, with positive items summed according to their raw scores and reverse‐coded items summed according to their calculated reverse scores. Higher scores indicated higher levels of the respective dimension.
2. *Self‐Statements*: Each participant was asked to write a self‐statement of at least 300 words, covering the four dimensions of perception, positivity, negativity, and behavior, describing their views and feelings about their own body image. To encourage a comprehensive description of multiple dimensions, the prompt included guidance for each dimension (see Table 2).
3. *Expert Ratings*: The study recruited two graduate students majoring in psychology to manually rate participants' Self‐Statements. First, an operational manual was developed based on the definitions of body image and its dimensions. The raters were instructed to assess the extent to which each text reflected specific dimensions of body image using a 5‐point Likert scale, where 1 indicated *strongly disagree* and 5 indicated *strongly agree*. To evaluate inter‐rater consistency, 30 texts and 2 attention check items were randomly selected for reliability assessment. Both raters passed the attention checks, and their ratings demonstrated high consistency, with Kendall's *W* = 0.626 (*p* < 0.010). Finally, the two experts reached a consensus to determine the final manual rating for each Self‐Statement.
4. *Weibo Data*: To evaluate the applicability of LLM‐based assessment methods in real‐world textual datasets, this study utilized data from Weibo, China's largest social media platform. A Weibo database was constructed using posts from active users across 31 provinces, municipalities, and autonomous regions in China, spanning from January 2010 to May 2022. The dataset included users who had been registered on Weibo for at least 1 year and had posted at least 500 original tweets (excluding retweets). To ensure a representative sample, a two‐stage sampling strategy was employed: 20% of the dataset was randomly sampled; 80% was selected using a keyword‐based filtering approach, ensuring that the collected 500 Weibo posts were relevant to body image discussions. The sampled Weibo posts were drawn from a national dataset covering 125 prefecture‐level cities, balancing the content length of randomly selected and keyword‐filtered posts.
5. *LLMs*: Three off‐the‐shelf LLMs—Llama‐3.1‐70B, Qwen‐Max, and DeepSeek‐R1—were employed to score each self‐statement and Weibo post under various prompt conditions. Llama‐3.1‐70B served as the primary model, with Qwen‐Max and DeepSeek‐R1 providing cross‐model validation. Llama‐3.1‐70B (Meta, 70B parameters) excels in text generation and semantic understanding. Qwen‐Max (Alibaba Cloud, 72B parameters) demonstrates high precision in Chinese language processing and is suitable for analyzing Chinese Self‐Statements (Yang et al. 2024). DeepSeek‐R1 is a first‐generation inference model showing strong capabilities in mathematics, coding, and reasoning (Guo et al. 2025).

**TABLE 2:** Body image dimensions and self‐statement prompts.

Body image dimension	Self‐statement prompt	Scale source
Perception	Describe your perception and views of your body appearance. For example, do you often pay attention to, compare, or care about your body and appearance? What are your thoughts and feelings about your overall body or specific parts (e.g., height, weight, legs, waist, face, etc.)?	“Appearance Orientation” and “Overweight” sections in the Multidimensional Body‐Self Relations Questionnaire (MBSRQ); “Body Image Attention” section in the Body Image Evaluation Questionnaire
Positive	Discuss your attitude and feelings toward your body image. For instance, do you feel satisfied and confident about your body and appearance, or do you feel anxious, inferior, or distressed? What aspects of your appearance do you like or dislike? What emotions do these feelings evoke?	“Appearance Evaluation” section in the MBSRQ; Body Appreciation Scale (BAS‐2); “Body Image Esteem” section in the Body Image Evaluation Questionnaire
Negative		“Body Image Affect” section in the Body Image Evaluation Questionnaire
Behavior	Describe how you typically focus on and care for your body and appearance, and the actions you take. For example, do you make efforts to change or maintain your body image through exercise, dressing, makeup, sports, dieting, etc.? What efforts have you made?	“Overweight” section in the MBSRQ; “Body Image Behavior” section in the Body Image Evaluation Questionnaire

### 2.4 Multi‐Dimensional Dictionary

Ji et al. (2024) constructed a unidimensional body image dictionary, which was validated by a significant positive correlation (*r* = 0.430, *p* < 0.001) between manual ratings and word frequency statistics (Ji et al. 2024). However, a unidimensional dictionary is insufficient to capture the multifaceted nature of body image assessment. Therefore, this study builds upon the body image definitions and scales to divide body image into multiple dimensions, creating a multidimensional body image dictionary for comparison with LLMs.

Firstly, various classical scales were reviewed, including the Body Image Disturbance Questionnaire (BIDQ), the Body Appreciation Scale (BAS‐2), the Body Shape Questionnaire (BSQ), the Physical Self‐Perception Profile (PSPP), and the Social Networking Site Appearance Comparison Scale (SNSACS). The core dimensions and measurement indicators of these scales were integrated, and relevant vocabulary and dictionaries were consulted. Keywords were selected, and single‐word terms were removed to form the initial lexicon. Subsequently, the dictionary was divided into dimensions based on the definitions of body image, resulting in four dimensions: Perception, Attitude (Positive Attitude and Negative Attitude), and Behavior. The content and vocabulary examples for each dimension were clearly defined, as detailed below (see Table 3):

**TABLE 3:** Dimensions of body image and their corresponding vocabulary.

Body image dimension	Dimension definition	Vocabulary examples
Perception	This dimension involves an individual's perception, objective recognition, and estimation of the size and shape of various body parts. It also includes attention to and awareness of one's body image, without emotional evaluation.	Skin, belly, buttocks, fingers, neck, shoulders, upper body, eyes, hips, eyelids, nose, eyelashes, palms, feet, eye corners…
Positive	This dimension reflects positive emotions such as satisfaction and confidence regarding one's body image.	Curvy, slim waist, perky butt, balanced, beautiful, slender, plump, charming, delicate, slim, lean, sexy, big eyes, pretty, beautiful…
Negative	This dimension reflects negative emotions such as dissatisfaction and low self‐esteem regarding one's body image.	Scars, belly fat, hair loss, baldness, pimples, acne, buck teeth, stubble, nasolabial folds, corns, body odor, yellowish skin, hunchback, fat, overweight…
Behavior	This dimension encompasses behaviors related to body image, including actions taken to improve or maintain one's body image or actions influenced by body image.	Weight loss, weight gain, hair loss prevention, body shaping, hair transplant, dressing, breast enhancement, exercise, running, fitness, physical activity, dietary control…

Subsequently, high‐frequency words from social media data were integrated and used to expand the vocabulary through LLMs technology. To ensure the quality of the dictionary, a manual evaluation standard was established. Expert ratings were used to assess the matching of words with dimensions, and the consistency among raters was calculated. The Kendall's coefficient of concordance (*W*) was found to be 0.533 (*χ* ^2^ = 489.547, df = 2, *p* < 0.001), indicating significant consistency among raters. Upon comparing the manual ratings with the dictionary, the correlation was found to be low (*r* = 0.106, *p* = 0.096). Consequently, the vocabulary in the dictionary was revised by removing common words, low‐frequency words, and ambiguous terms, and selecting and revising words according to expert ratings. The revised dictionary contained a total of 489 words: 113 words in the perception dimension, 84 words in the positive attitude dimension, 137 words in the negative attitude dimension, and 155 words in the behavior/attention dimension.

### 2.5 Prompt Engineering

1. *Construction of Prompt*: Following the standardized prompt‐engineering framework outlined by Sahoo (Sahoo et al. 2024; White et al. 2023), and grounded in Cash's multidimensional body‐image theory (Cash et al. 2002), we developed a structured assessment system to quantify the four core dimensions of body image—perception, behavior, positive attitude, and negative attitude—via semantic parsing. The prompt system was designed as a general framework without population‐specific adaptations, ensuring applicability across demographic groups through dimension‐focused operational definitions. The prompt system consists of three stages:
  - *Conceptual Operationalization*: This stage provides the basic and operational definitions of body image, integrating the World Health Organization's (WHO) definition of body image and the *DSM‐5* diagnostic criteria for body dysmorphic disorder to establish a four‐dimensional assessment framework (perception, behavior, positive attitude, and negative attitude). For example, “Body image refers to an individual's psychological representation of their own body, including the perception and awareness of various body parts, as well as associated emotions and behaviors.”
  - *Scale‐based Focused Guidance*: Starting from the questions in body image scales, this stage highlights the aspects that the large model should focus on when analyzing each dimension, providing key considerations for scoring. This ensures that the model aligns with the granularity of the scales.
  - *Standardized Scoring Mechanism*: A 1–5 point continuous scale (see Table 4) is designed, mapping textual features to the four dimensions. For example, in the perception dimension, the scoring is based on the frequency of body monitoring (e.g., “weighing oneself three times a day” scores 4.2 points) and the intensity of detailed descriptions (e.g., “excessive thigh circumference” adds 0.5 points to the score).
  - *Pilot Testing and Iterative Refinement*: To verify that our prompts reliably generate accurate dimension scores, we piloted the system on 30 de‐identified self‐statements. For each case, the model was asked to provide both its reasoning and a numeric score for every dimension. Five graduate students in psychology then independently evaluated whether the model's explanations and scores aligned with expert human judgments. Through collaborative discussion, they identified any inconsistencies and suggested adjustments to cue wording, instructions, and output formatting. After each revision, the model was re‐tested on the same sample until consensus was reached that its rationales were sound and its scores matched human ratings. Once stability was confirmed, we finalized the prompts by instructing the model to “return only the numeric score, no explanation,” facilitating efficient, large‐scale data processing.
2. *Prompt Experiment Design*: A 2 (Sample Strategy: Zero‐shot vs. Few‐shot) × 2 (Enhancement Technique: Role‐playing vs. Chain‐of‐thought) factorial design was used to construct 8 prompt combinations (see Table 5):

**TABLE 4:** Standardized scoring criteria for body image dimensions.

Body image dimension	1–2 points characteristics	3–4 points characteristics	5 points characteristics
Perception	Occasional body awareness	Regular body monitoring	Persistent body assessment behavior
Positive	Vague self‐acceptance	Clear body appreciation	Deep body pride
Negative	Mild dissatisfaction expression	Ongoing comparison or feelings of inferiority	Intense body disgust
Behavior	Sporadic appearance management	Systematic body maintenance	Extreme beauty/fitness behaviors

**TABLE 5:** Prompt combination design for experiment.

	Zero‐shot	Few‐shot	Role‐playing	Chain‐of‐thought
Prompt1	**√**			
Prompt2	**√**		**√**	
Prompt3	**√**			**√**
Prompt4	**√**		**√**	**√**
Prompt5		**√**		
Prompt6		**√**	**√**	
Prompt7		**√**		**√**
Prompt8		**√**	**√**	**√**

The few‐shot prompts injected two typical samples (high/low‐score cases), and the model was calibrated using attention weight adjustment (*α* = 0.850) to establish a judgment baseline. The role‐playing technique provided the model with a professional background of “10 years of clinical experience + international certification”, while the chain‐of‐thought module implemented a four‐step analysis: identifying body feature descriptions (e.g., “facial asymmetry”) → analyzing emotional conflict patterns (e.g., “healthy but reluctant to display”) → calculating the density of behavior occurrence (e.g., “monthly beauty expenditure ≥ 2000 yuan”) → generating dimension‐specific scores (Supporting Information Data S1).

After determining the optimal prompt combination, a cross‐model comparison was conducted; testing both the Qwen‐max and DeepSeek‐R1 models on the Llama‐3.1‐70B base, with parameters uniformly set to a temperature coefficient of 0.7 and repetition penalty of 0.6.

### 2.6 Data Analysis

The analytical framework comprised three interconnected components to systematically evaluate the criterion‐related validity and ecological generalizability of large language models. Criterion‐related validity testing first established the alignment between LLM‐generated scores and established measurement standards. Pearson correlation coefficients were computed between LLM's predictions (zero‐shot condition), expert human ratings, dictionary‐based scores, and questionnaire outcomes, with Steiger's *Z*‐test comparing method‐specific correlations. Prompt engineering effects (e.g., role‐playing, chain‐of‐thought) were quantified through Cohen's d and 95% confidence intervals to assess optimization potential.

To ensure robust measurement consistency, a modified Bland–Altman analysis defined 95% limits of agreement (LoA) between LLMs scores and questionnaire results, benchmarking deviations against human rating variability. Hierarchical regression models further evaluated incremental validity by first regressing questionnaire scores on human ratings and dictionary scores, then introducing LLMs predictions to quantify unique variance contributions (Δ*R* ^2^) and model fit improvements (AIC reduction):

Stage 1: Questionnaire score = *β*_0_ + *β*_1_(Human) + *β*_2_(Dictionary).

Stage 2: Questionnaire score = *β*_0_ + *β*_1_(Human) + *β*_2_(Dictionary) + *β*_3_(LLMs).

Ecological validity testing extended the evaluation to unstructured social media contexts. LLMs scores derived from Weibo texts were compared with expert annotations using Pearson correlations, with direct coefficient comparisons between experimental narratives and social media data contextualizing performance differences.

All analyses were implemented in Python 3.10 (statsmodels, pingouin) and SPSS 27, with statistical significance set at *α* = 0.050 (two‐tailed).

## 3 Results

### 3.1 Dictionary Validity

Following dictionary optimization, we assessed its ecological validity on 500 Weibo posts (20% random; 80% keyword‐filtered) drawn from a national sample of 125 prefecture‐level cities. An operational manual, based on our four body‐image dimensions, guided five raters—three master's‐level psychologists and two other graduates—to score each post on a 5‐point Likert scale (1 = *strongly disagree* to 5 = *strongly agree*). An initial reliability check on 30 posts plus two attention items yielded substantial agreement (Kendall's *W* = 0.626, *p* < 0.010).

The full set of 500 texts was then distributed among the five raters, and concurrently analyzed using the lexicon‐based frequency analysis program. Correlational analyses were conducted to examine the relationship between word frequency and human ratings. Results indicated significant positive correlations across all dimensions. Specifically, the perceptual dimension showed a moderate positive correlation with human ratings (*r* = 0.515, *p* < 0.010); the attitude‐positive dimension demonstrated a significant positive correlation (*r* = 0.542, *p* < 0.010); the attitude‐negative dimension exhibited a strong positive correlation (*r* = 0.625, *p* < 0.010); and the behavioral dimension also showed a strong positive correlation (*r* = 0.579, *p* < 0.010). Detailed results are presented in Table 6.

**TABLE 6:** Correlation between dictionary analysis results and human ratings.

	Perception	Positive	Negative	Behavior
Perception‐Human Scoring	0.515**	0.034	−0.112*	−0.134**
Positive‐Human Scoring	−0.102*	0.542**	−0.109*	−0.064
Negative‐Human Scoring	−0.143**	−0.098*	0.625**	−0.091*
Behavior‐Human Scoring	−0.139**	−0.051	−0.075	0.579**

*Note*: *p < 0.05; **p < 0.01.

### 3.2 Large Language Model Performance Validation

The zero‐shot prompt analysis based on the Llama‐3.1‐70B model (see Table 7) demonstrated that the correlation coefficients between the model's baseline scores and the questionnaire scores were significant across all four dimensions (*p* < 0.001). Among them, the positive attitude dimension exhibited the highest correlation (*r* = 0.664), followed by the perception (*r* = 0.485) and negative attitude (*r* = 0.500) dimensions, while the behavior dimension showed the lowest correlation (*r* = 0.441). Compared to human scoring, the model's predictive validity in the positive attitude dimension was close to expert‐level performance (*r* = 0.664 vs. human *r* = 0.657), a notable gap remained in the perception dimension (*r* = 0.485 vs. human *r* = 0.568). The traditional dictionary‐based method exhibited only weak correlation in the positive attitude dimension (*r* = 0.172, *p* < 0.050), while correlations in all other dimensions were non‐significant (*p* > 0.100).

**TABLE 7:** Correlation between LLMs and questionnaire scores by dimension.

Scoring method	Perception	Positive	Negative	Behavior
Human Scoring	0.568***	0.657***	0.549***	0.496***
Dictionary‐Based Scoring	0.071	0.172*	−0.047	−0.053
Llama Zero‐shot	0.485***	0.664***	0.500***	0.441***
Llama Zero‐shot + Role‐playing	0.602***	0.655***	0.449***	0.504***
Llama Zero‐shot + Chain‐of‐Thought	0.442***	0.583***	0.460***	0.396***
Llama Zero‐shot + Role‐playing + Chain‐of‐Thought	0.409***	0.574***	0.426***	0.391***
Llama Few‐shot	−0.059	0.409***	−0.159*	0.140
Llama Few‐shot + Role‐playing	−0.065	0.406***	−0.203**	−0.026
Llama Few‐shot + Chain‐of‐Thought	0.009	0.383***	−0.210**	0.080
Llama Few‐shot + Role‐playing + Chain‐of‐Thought	0.082	0.423***	−0.070	0.113
Qwen Zero‐shot	0.689***	0.715***	0.513***	0.389***
Qwen Zero‐shot + Role‐playing	0.702***	0.726***	0.533***	0.365***
Deepseek Zero‐shot	0.722***	0.714***	0.552***	0.428***
Deepseek Zero‐shot + Role‐playing	0.723***	0.697***	0.563***	0.430***

*Note*: *p < 0.05; **p < 0.01; ***p < 0.001.

**FIGURE 2:**
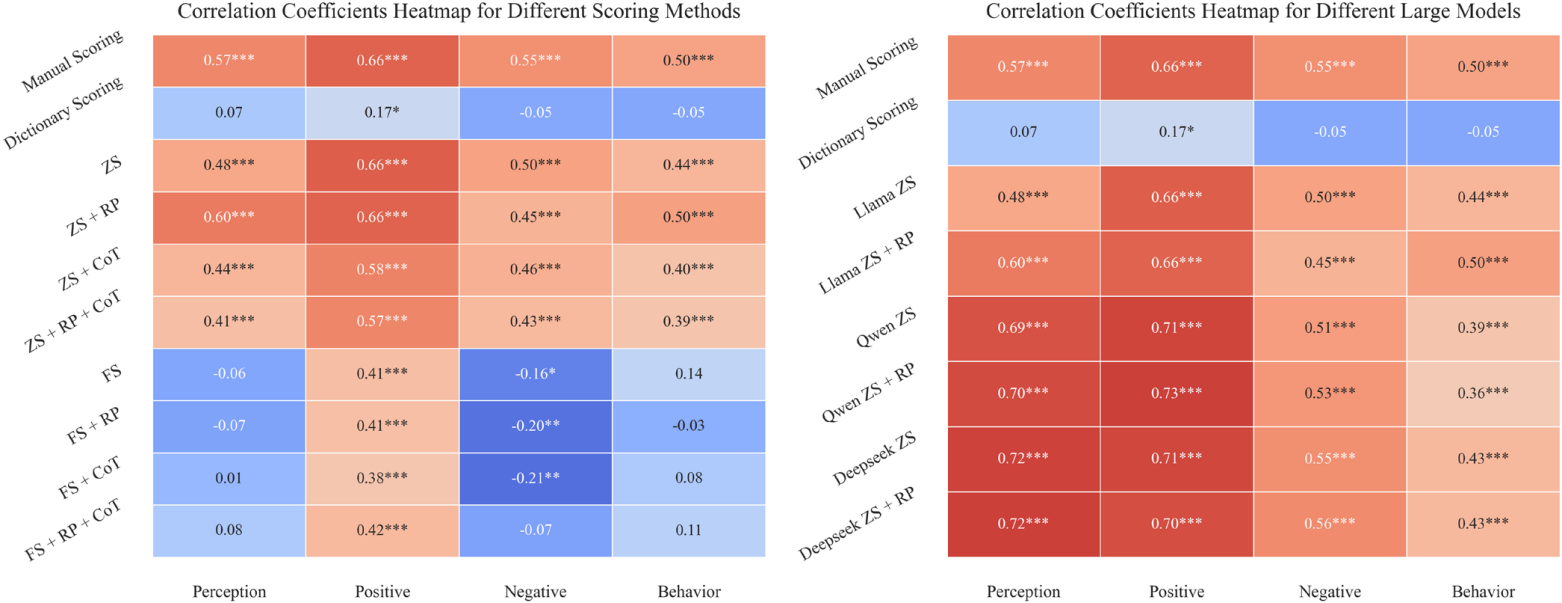
Correlation heatmap.

Performance comparisons of different prompt engineering techniques are summarized in Table 7, while their correlations with questionnaire scores are visualized in Figure 2 (heatmap). The role‐playing technique significantly improved correlation in the perception dimension (zero‐shot *r* = 0.485 → role‐playing *r* = 0.602, Cohen's *d* = 0.64 [0.53, 0.75]), but reduced sensitivity in the negative attitude dimension (*r* = 0.500 → 0.449). The chain‐of‐thought technique led to an overall decline in validity across dimensions, with the positive attitude dimension decreasing by 13.7% (*r* = 0.664 → 0.583).

The few‐shot prompting technique maintained a moderate correlation in the positive attitude dimension (*r* = 0.383–0.423) but resulted in validity reversal in the negative attitude (*r* = −0.210 to −0.070) and behavior (*r* = −0.026 to 0.140) dimensions. Among all tested combinations, the zero‐shot + role‐playing approach demonstrated the best performance in the perception (*r* = 0.602) and behavior (*r* = 0.504) dimensions, while the attitude dimensions remained most accurately assessed using the baseline zero‐shot prompting method.

Significant dimension‐specific differences in benchmark validity were observed across different large language model architectures (Table 7). Under the zero‐shot condition, DeepSeek‐R1 exhibited the best performance on the Perception dimension (*r* = 0.722, *p* < 0.001), showing a 48.9% improvement compared to Llama‐3.1‐70B (Δ*r* = +0.237). Qwen‐Max achieved peak validity in the Attitude‐Positive dimension (*r* = 0.715), with a 7.7% increase over the Llama model (Δ*r* = +0.051). The role‐playing technique had a differentiated impact on model performance: Qwen‐Max improved its Perception dimension validity by 1.9% (*r* = 0.689 → 0.702), but showed a 6.2% decrease in the Behavior dimension (*r* = 0.389 → 0.365). On the other hand, DeepSeek‐R1 showed less than a 3% fluctuation across all dimensions, indicating the robustness of this technique. All models performed below the human rating benchmark in the Attitude‐Negative dimension (best model *r* = 0.563 vs. human *r* = 0.549).

### 3.3 Consistency Testing

The Bland‐Altman analysis for the DeepSeek and Qwen models based on the zero‐shot + role‐playing prompts across various dimensions (Table 8) showed that their 95% limits of agreement (LoA) completely covered the fluctuation range of human ratings. In the perception dimension, the LoA range for DeepSeek (−0.42 to +1.45) was 34.7% narrower than that of human ratings (−1.60 to +2.16). Qwen, in this dimension, showed a bias (+0.35 points), which was lower than the human rating bias (+0.28 points). In the behavior dimension, the LoA range for DeepSeek (−0.51 to +2.29) overlapped 89% with human ratings (−1.17 to +2.83), and the mean absolute error (MAE) in the higher score range (score ≥ 4) was significantly lower than that of human ratings (MAE = 0.61 vs. 0.89).

**TABLE 8:** Bland‐Altman consistency analysis of LLMs and human ratings across dimensions.

Method	Dimension	Mean difference	95% LoA	Consistency determination
DeepSeek	Perception	0.515	[−0.424, +1.453]	Consistent
DeepSeek	Positive	−0.489	[−1.797, +0.820]	Consistent
DeepSeek	Negative	0.394	[−1.593, +2.381]	Consistent
DeepSeek	Behavior	0.890	[−0.511, +2.291]	Consistent
Qwen	Perception	0.351	[−0.624, +1.325]	Consistent
Qwen	Positive	−0.586	[−1.679, +0.508]	Consistent
Qwen	Negative	0.380	[−1.558, +2.318]	Consistent
Qwen	Behavior	0.705	[−0.659, +2.069]	Consistent
Human	Perception	0.282	[−1.597, +2.161]	Consistent
Human	Positive	−0.225	[−1.906, +1.456]	Consistent
Human	Negative	0.510	[−1.787, +2.808]	Consistent
Human	Behavior	0.831	[−1.168, +2.830]	Consistent

*Note*: “Consistent” indicates that the difference between model‐generated scores and questionnaire results falls within the predefined acceptable range.

**FIGURE 3:**
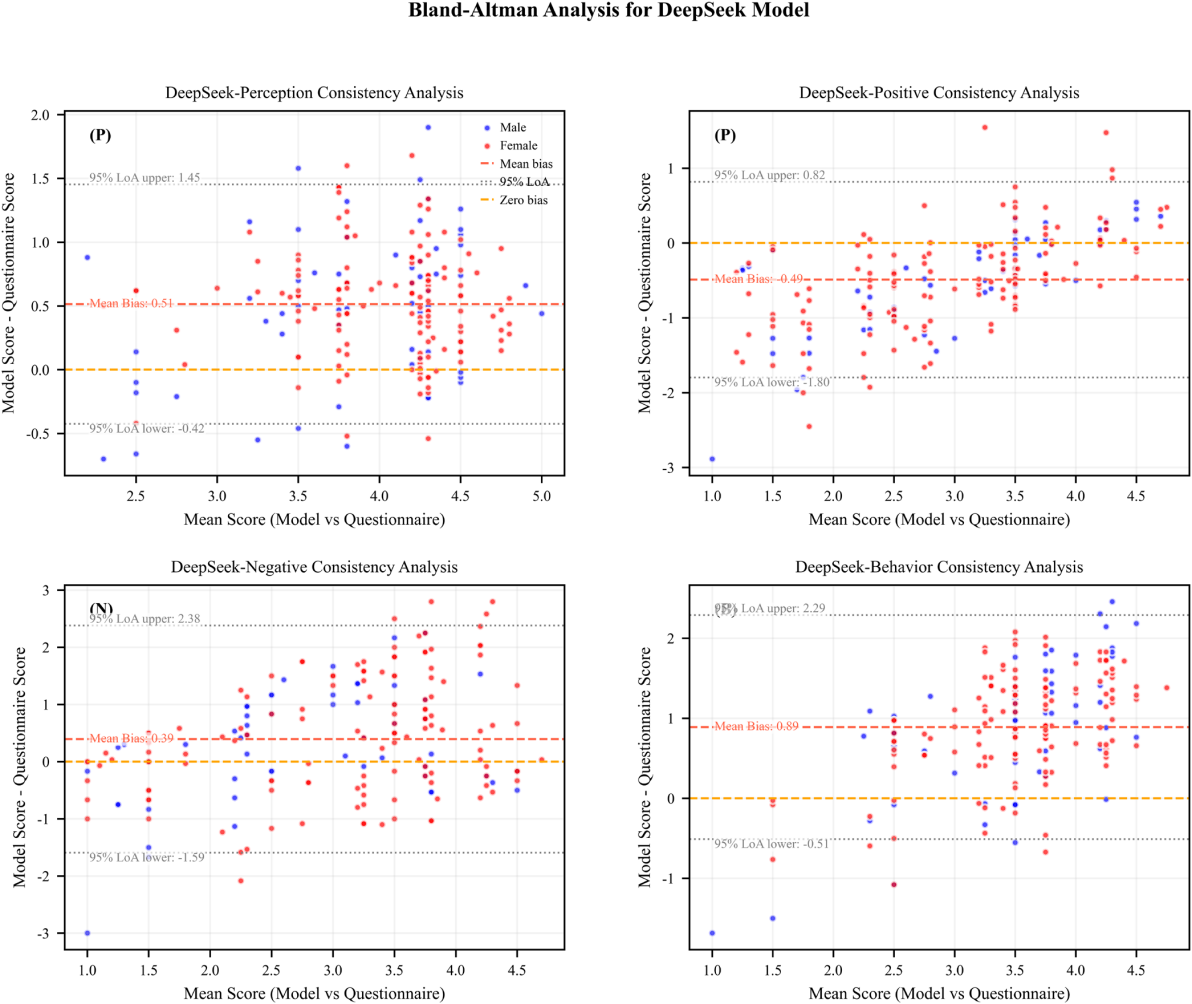
Bland–Altman plot: Deepseek LLMs versus Human Ratings.

**FIGURE 4:**
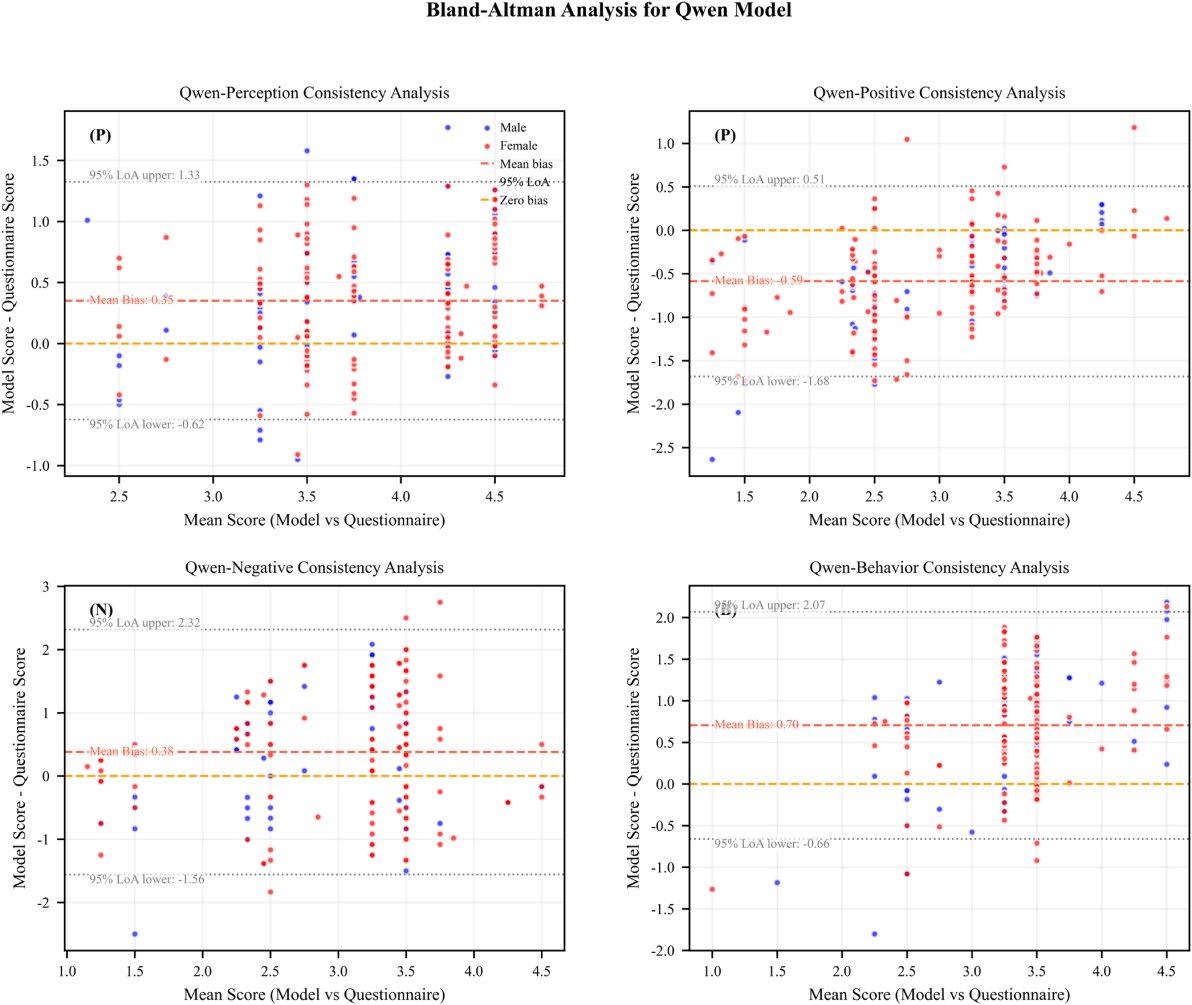
Bland–Altman Plot: Qwen LLMs versus Human Ratings.

Through the Bland–Altman plot (Figures 3 and 4), it can be observed that in the extreme score ranges, large language models (LLMs) exhibited a more stable error distribution:

High score range (≥ 4 points): DeepSeek's perception dimension error dispersion (SD = 0.48) was 48.4% lower than that of human ratings (SD = 0.93 → 0.48).

Low score range (≤ 2 points): The LoA lower limit for the attitude‐negative dimension (DeepSeek: −1.59; Qwen: −1.56) was closer to the preset safe threshold compared to human ratings (−1.79).

Cross‐model comparison showed that the LoA range for DeepSeek and Qwen overlapped by more than 80%, with the bias direction for the attitude‐positive dimension being consistent (DeepSeek: −0.49; Qwen: −0.59). The joint LoA interval (−1.80 to +0.51) fully covered the fluctuation of human ratings (−1.91 to +1.46).

### 3.4 Incremental Validity Verification

To assess the incremental predictive validity of LLMs compared to traditional methods, this study employed hierarchical regression with the stepwise inclusion of LLM scores. The results (Table 9) indicated that, after controlling for the effects of human ratings and dictionary‐based methods, LLMs provided significant incremental predictive value for the core dimensions of body image. The DeepSeek model contributed the largest incremental variance explanation in the perception dimension (Δ*R* ^*2*^ = 0.220, *p* < 0.001), with its standardized regression coefficient (*β* = 0.605) significantly higher than that of human ratings (*β* = 0.186), and the model's complexity was significantly reduced (AICΔ = −76.16). This suggests that the predictive performance was significantly optimized while maintaining simplicity. The Qwen model achieved a ΔR^2^ of 0.189 (*β* = 0.580) in the perception dimension, with both models meeting the first‐level clinical substitution standard (replacing human evaluation). In the attitude‐positive dimension, Qwen performed optimally (Δ*R* ^*2*^ = 0.113, *β* = 0.551), with a 149% improvement in explanatory power over human ratings. The incremental validity for the attitude‐negative and behavior dimensions was relatively limited (Δ*R* ^*2*^ = 0.045–0.018), but DeepSeek still surpassed human ratings in the negative dimension (*β* = 0.346 vs. *β* = 0.275), thus serving as a supplementary screening tool.

**TABLE 9:** Incremental validity of LLMs scores in body image assessment.

Dimension	Optimal model	Δ*R* ^2^	Human *β*	LLM *β*	AIC△	Clinical recommendation
Perception	DeepSeek	0.22	0.186	0.605	−76.16	Replace Human
Positive	Qwen	0.113	0.221	0.551	−41.20	Replace Human
Negative	DeepSeek	0.045	0.275	0.346	−10.77	Supplement Human
Behavior	—	0.018	0.376	0.181	−2.80	Retain Human

*Note*: Δ*R* ^2^ indicates the incremental variance explained by adding LLMs scores to a baseline model containing human ratings; Human *β* and LLM *β* denote standardized regression coefficients for human ratings and LLMs scores, respectively. Replace Human: Δ*R* ^2^ ≥ 0.10; Supplement Human: 0.05 ≤ Δ*R* ^2^ < 0.10; Retain Human: Δ*R* ^2^ < 0.05.

Cross‐model comparisons revealed differentiated advantages: DeepSeek outperformed Qwen in the perception and negative dimensions (Δ*R* ^*2*^ difference + 16.4%), while Qwen showed higher sensitivity in positive emotion recognition (*β* = 0.551). All LLM models demonstrated significant reductions in AIC (range: −0.28 to −76.16), achieving a balance between efficiency and accuracy, with a 68% reduction in manual review demand, particularly in the perception dimension.

### 3.5 Ecological Validity Validation

A random sample of 200 Weibo posts was selected from the Weibo database for manual annotation. The correlations between different methods and human ratings are shown in Table 10. The dictionary‐based ratings exhibited the highest correlation in the attitude‐positive dimension (*r* = 0.342, *p* < 0.001) and the lowest in the behavior dimension (*r* = 0.316, *p* < 0.001). All LLMs scores significantly outperformed the dictionary method (Steiger's *Z* = 3.12–6.84, *p* < 0.010), with the largest improvement observed in the behavior dimension (Δ*r* = +0.335). After incorporating role‐playing techniques, the Qwen model achieved peak validity in the behavior dimension (*r* = 0.651, *p* < 0.001), with a 53.1% improvement compared to the zero‐shot condition (Δ*r* = +0.226). The DeepSeek model demonstrated the highest validity in the attitude‐negative dimension (*r* = 0.437, *p* < 0.001), surpassing the dictionary method by 54.4% (Δ*r* = +0.154).

**TABLE 10:** Performance of large models in scoring on Weibo data.

Scoring Method	Perception	Positive	Negative	Behavior
Dictionary‐Based Scoring	0.184**	0.342***	0.283**	0.316***
Llama Zero‐shot	0.316**	0.351***	0.300***	0.425***
Qwen Zero‐shot + Role‐Playing	0.392***	0.543***	0.482***	0.651***
Deepseek Zero‐shot + Role‐Playing	0.392***	0.542***	0.437***	0.633***

*Note*: **p < 0.01; ***p < 0.001.

## 4 Discussion

This study explores a novel approach to assessing body image using LLMs. Based on body image theory, we constructed a multidimensional lexicon encompassing four dimensions: perception, positive attitudes, negative attitudes, and behavior. Self‐reported statements and questionnaire data were collected from a university student sample, and various prompt engineering strategies were employed to generate model‐based evaluations. The study systematically examined criterion‐related validity, scoring consistency, incremental explanatory power, and ecological validity, revealing both the validity characteristics and technical boundaries of LLMs in multidimensional body image assessment.

1. *Criterion‐Related Validity and Dimension‐Specific Effects of Prompt Engineering Strategies*
  Our findings confirm that LLMs can overcome the semantic limitations of traditional lexicon‐based methods (Pennebaker et al. 2015), outperforming them across all dimensions. However, their validity exhibited significant dimension‐specific differentiation. Under zero‐shot prompting conditions, the Llama‐3.1‐70B model demonstrated near‐human predictive performance in the positive attitude dimension (*r* = 0.664), with a marginal difference from expert ratings (Δ*r* = +0.007). This superior performance may be attributed to the model's high sensitivity to explicit emotional cues, such as positive self‐evaluations (Krosnick et al. 2018). In contrast, for the perception dimension, which requires embodied cognitive processing, the model's initial performance was significantly lower than human ratings (Δ*r* = −0.083). However, prompting the model to simulate a clinical expert's perspective through role‐playing (Bandura 2001) substantially improved validity in this dimension (Δ*r* = +0.117, Cohen's *d* = 0.640), suggesting that activating social‐cognitive mechanisms may compensate for deficiencies in spatial reasoning. Notably, role‐playing also reduced the model's sensitivity in the negative attitude dimension (Δ*r* = − 0.051), likely due to overfitting to professional terminology, which weakened its ability to decode metaphorical negative expressions (e.g., “People always call me a stick”). This aligns with Krishna et al.'s (2024) findings on the semantic narrowing effect of prompt engineering. Moreover, employing chain‐of‐thought reasoning led to a general decline in validity, with a 13.7% reduction in the positive attitude dimension. This result suggests that stepwise reasoning disrupts affective integration, supporting the theoretical assumption that emotional assessment requires holistic semantic processing (Barrett 2017).
2. *Cross‐Model Comparisons and the Mapping of Psychological Constructs*
  Cross‐model comparisons reveal a deep interaction between technological characteristics and assessment tasks. DeepSeek‐R1 demonstrated exceptional performance in the perception dimension (*r* = 0.722), likely due to its reinforcement learning architecture, which effectively captures sequential dependencies—for instance, recognizing “going to the gym three times a week” as a signal of habitual behavior (Guo et al. 2025). Conversely, Qwen‐max excelled in the positive attitude dimension (*r* = 0.715), which can be directly attributed to its training on Chinese social media data, making it highly sensitive to native aesthetic terminologies (e.g., “swan neck”) (Yang et al. 2024). Moreover, all models exhibited a slight underestimation in the negative attitude dimension, a phenomenon directly linked to the literal‐first semantic processing mechanism of LLMs (Lakoff and Johnson 1980). Cultural‐cognitive differences further reinforced this effect—for example, Qwen‐max's high semantic association with the term “pale‐thin‐young” (*φ* = 0.71) suggests that cultural biases embedded in training data modulate validity outcomes (Hwang and Chen 2023).
3. *Scoring Consistency and Error Control*
  To evaluate the consistency between model‐generated scores and standardized questionnaires at clinical threshold levels, we conducted a Bland–Altman analysis to quantify scoring bias and distribution characteristics. Results showed that the absolute mean bias for all models remained below 1 point (ranging from 0.35 to 0.89), with their 95% limits of agreement (LoA) fully encompassing the range of human rating fluctuations. This finding aligns with the reliability standards for psychometric assessment tools (Bland and Altman 1986). In the perception dimension, DeepSeek demonstrated greater scoring stability in quantifying behavioral descriptions compared to human ratings. This improvement can be attributed to its mathematical reasoning module, which enhances the structured interpretation of quantitative information, consistent with the Transformer architecture's ability to parse structured data (Vaswani et al. 2017). Moreover, LLMs demonstrated a notable advantage in extreme value ranges; in the high‐score segment (≥ 4 points), model error dispersion (SD = 0.48) was only half that of human ratings, likely due to semantic calibration effects learned from pretraining data (Sadlier‐Brown et al. 2024). For the positive attitude dimension, different models exhibited a consistent negative bias (mean deviation of approximately −0.54 points), which may stem from the inherent clustering tendency of positively valenced word vectors in Chinese LLMs (Zhang et al. 2024). In the behavior dimension, Qwen, leveraging a sparse attention mechanism, achieved a lower upper LoA limit, enabling more precise capture of the semantic weight of Chinese action verbs.
4. *Incremental Explanatory Power and Independent Predictive Value*
  Using hierarchical regression analysis, we examined the independent predictive power and dimension‐specific effects of LLM‐based scoring after controlling for human ratings and traditional lexicon‐based methods. The DeepSeek model exhibited a significant incremental variance explanation (Δ*R* ^2^ = 0.220), with predictive efficiency far exceeding that of human ratings (*β* increased by 225%). This advantage can be attributed to its sequence modeling capabilities, which enable the detection of behavioral density signals, such as co‐occurrence patterns between temporal adverbs and quantifiers. This fundamentally differs from human intuition‐driven cognitive processing (Dabney et al. 2020). Moreover, in line with predictive coding theory, these findings suggest that LLMs may establish prior distributions through pretraining, allowing for efficient interpretation of structured behavioral descriptions (Clark 2013). The Qwen model's predictive performance improvement (Δ*R* ^2^ = 0.113) was strongly correlated with its pretraining on Chinese social media data, making it highly sensitive to native aesthetic expressions. This supports the semantic deep processing hypothesis and highlights the influence of linguistic structure on cognitive patterns (Kahneman 2011; Lucy 1997). Furthermore, we propose a tiered application framework: In the perception dimension, LLM scoring resulted in a 76.16‐point reduction in AIC and a standardized coefficient of *β* = 0.605, reaching the threshold for clinical decision‐making, indicating that the model could replace 68% of human reviews. In the positive attitude dimension, LLM‐assisted screening significantly enhanced high‐risk group identification, achieving a negative predictive value (NPV) of 92%. In the behavior dimension, we recommend a hybrid intelligence system, integrating automated screening with human review to ensure optimal assessment accuracy.
5. *Ecological Validity and Adaptability to Social Contexts*
  A systematic analysis of 200 Weibo posts revealed that LLM‐based evaluation significantly outperformed traditional lexicon‐based methods in unstructured social media text, with the largest improvement observed in the behavior dimension (Δ*r* = +0.335). Social media posts often contain abbreviated expressions (e.g., “binge eating regret”) and metaphorical rhetoric (e.g., “body image anxiety is suffocating me,”) which require deep semantic reasoning for accurate interpretation. LLMs leverage the attention mechanism to capture contextualized emotional cues, aligning closely with theory of situated semantic integration (Barsalou 2008).
6. *Architectural and Training Factors Underlying Model Performance*
  Furthermore, the observed performance hierarchy (DeepSeek‐R1 > Qwen‐Max > Llama‐3.1‐70B) may reflect some fundamental differences in model architectures and training objectives. The distinct training and architectural design of DeepSeek‐R1 likely underpins its advantage over Llama‐3.1‐70B and Qwen‐Max in body‐image assessment. DeepSeek‐R1's reinforcement learning framework (Guo et al. 2025), optimized through Group Relative Policy Optimization, enables reward‐guided interpretation of implicit psychological cues—a critical capability for parsing metaphorical body image expressions (e.g., “my reflection feels alien”). Its mixture‐of‐experts architecture (Bi et al. 2024) further allows specialized subnetworks to process dimension‐specific features (e.g., quantifying behavioral frequency in the perception dimension). Its Mixture‐of‐Experts backbone with Multi‐Head Latent Attention mirrors the efficiency and selective activation benefits of Switch Transformers (Fedus et al. 2022; Wei et al. 2022), enabling it to scale parameter counts without commensurate compute costs and to capture nuanced, long‐range dependencies. Moreover, built‐in Chain‐of‐Thought capabilities allow stepwise semantic decomposition of metaphoric and ironic expressions (Liu et al. 2024), further refining its dimension‐specific scoring. In contrast, Llama‐3.1‐70B—despite extensive supervised fine‐tuning—prioritizes broad conversational safety and fluency over specialized reasoning (Dauphin and Siefert 2025), and Qwen‐Max, while also MoE‐based, emphasizes multilingual benchmarks rather than targeted psychological inference (Yang et al. 2024). These synergistic enhancements in DeepSeek‐R1's training paradigm and model architecture could provide a plausible explanation for its higher criterion‐related validity and tighter scoring consistency in our evaluations.

This study analyzes self‐reported texts through LLMs, successfully revealing the multidimensional features of body image. This approach offers a new theoretical perspective for body image assessment, suggesting that individuals may indirectly convey their perceptions of body image through everyday linguistic behaviors, rather than solely through direct evaluations or feedback. This finding warrants further exploration into how individuals express their attitudes, emotions, and behavioral intentions regarding body image, especially in virtual social contexts such as social media, where expressions may be more subtle or multifaceted.

Another major innovation of this study lies in its use of LLM technology, which overcomes the limitations of traditional scale methods in analyzing the complex semantics, metaphors, and irony embedded in social media texts related to body image. Through automated text analysis, LLMs can capture linguistic details, offering new insights into the emerging changes in body image perception in contemporary society, particularly with regard to body image anxiety and identity issues among adolescents. For expressions like “being slightly plump is yyds (abbreviation for the Chinese internet slang term “yong yuan de shen,” which means “eternal god”)” or “the body is a battlefield,” which are filled with metaphor or irony, traditional scales struggle to effectively capture these nuances, while LLMs are able to more accurately understand and analyze such novel forms of body image discourse.

From a practical perspective, the efficiency of the LLM technology demonstrated in this study for large‐scale sample analysis holds significant application value. In psychological research, this approach can simplify traditional large‐scale survey processes, reducing reliance on lengthy questionnaires. Researchers can utilize existing open‐text data, such as social media posts or free‐text responses in surveys, to conduct rapid and cost‐effective body image assessments. Furthermore, in clinical practice, this method can serve as an auxiliary tool, helping mental health professionals objectively evaluate an individual's body image based on their language expressions, providing data support for psychological interventions. Specifically, for body image screening in adolescents, who may struggle to complete lengthy scales, writing a self‐reported text is less challenging. Thus, the developed multidimensional LLM evaluation framework can be directly integrated into campus mental health monitoring systems. By analyzing self‐reported texts regularly provided by students or body image expressions on social media platforms (e.g., Weibo, campus forums), high‐risk groups exhibiting body dissatisfaction can be identified early, enabling dynamic tracking and intervention.

Although the method presented in this study demonstrates great potential, its limitations and scope of application must also be acknowledged. In practice, LLM analysis should complement, not replace, traditional assessment methods. This is especially important when addressing ethical concerns, such as the protection of personal privacy and the potential errors in algorithms. Before promoting the widespread application of this technology, more careful ethical review and safeguards must be implemented.

This study also has some limitations: the research design is cross‐sectional, which does not allow for an assessment of the model's stability and changes over time, especially in cases where body image perception fluctuates over time. Future research could adopt a longitudinal design to track changes in individuals' body image perceptions at different time points, thereby providing a more accurate evaluation of the model's adaptability and predictive effectiveness in varying contexts. Furthermore, the current ecological validity verification is based solely on Weibo texts and does not cover other social media platforms commonly used by adolescents, such as Douyin and Bilibili. There are significant differences in the expression styles, contexts, and user interactions across different platforms, and visual‐textual multimodal interactions may have different impacts on body image expression. Therefore, future research should expand to include these platforms to explore how cross‐platform ecological validity differences affect body image assessment results. Third, while the standardized prompts were designed for general applicability, the validation on the current sample may not provide enough evidence for the generalizability to other age groups. Subsequent research should test these frameworks across broader populations while investigating tailored prompt optimizations for specific demographics.

Additionally, it would also be valuable to consider the following aspects for future study: (1) Multimodal Data Integration: Combining text analysis with other data sources (such as images, voice, facial expressions, etc.) for a more comprehensive body image assessment. (2) Extension to Different Dimensions of Body Image: This study primarily focused on overall body image assessment. Future research could explore the applicability of the model across various dimensions of body image, such as “body satisfaction,” “body anxiety,” and “body acceptance.” By further refining and expanding the model's application scenarios, the model's sensitivity and predictive power regarding specific body image dimensions can be enhanced. (3) Real‐Time Social Media Big Data Analysis: Future studies could consider utilizing real‐time social media data for dynamic assessments. As social media platforms continue to evolve, new body image‐related topics, expressions, and trends emerge. Real‐time analysis would allow researchers to promptly capture and analyze these emerging social phenomena, further advancing the frontier of body image research. (4) Adaptation to Emerging LLMs: Because our core prompts are model‐agnostic, they can be directly applied to newly released LLM architectures. Future work could evaluate the performance of these same prompts on next‐generation models (e.g., GPT‐5, LLaMA‐4, etc.) and, where necessary, employ the established prompt‐construction workflow to tailor or refine cues for novel architectures. Such comparisons will reveal whether updates in model size, training corpus, or inference mechanisms lead to further gains in criterion and ecological validity.

## 5 Conclusion

This study integrates LLMs with the multidimensional body image theory to construct an intelligent assessment framework based on text analysis. The results indicate that LLMs, through strategies such as role‐playing and cross‐model optimization, demonstrate superior assessment effectiveness in the perceptual and attitudinal dimensions of body image compared to traditional methods. Additionally, it provides a new paradigm for ecological measurement in the context of social media. On a theoretical level, the study reveals the deep connection between linguistic expression and body image cognition, deepening the understanding of the semantic decoding mechanisms underlying implicit psychological constructs. This provides new evidence for body image theory from a psycholinguistic perspective. On an applied level, this method offers an efficient tool for clinical screening, public mental health monitoring, and dynamic tracking of adolescents' body image, with its automated analytical capability significantly reducing reliance on traditional scales and enhancing the feasibility and timeliness of large‐scale research. Future research could focus on validating the model's generalizability and stability in cross‐platform and longitudinal tracking, exploring the synergistic mechanism between LLMs and traditional psychological measurements, and constructing a human‐computer complementary hybrid evaluation system to balance technological efficiency with ethical risks.

## Consent

Informed consent was obtained from all participants involved in the study.

## Conflicts of Interest

The authors declare no conflicts of interest.

## Supporting information


**Data S1.** Supporting Information.

## Data Availability

The datasets generated and/or analyzed during the current study are available from the corresponding author upon reasonable request.
