# Supplementary material for: The Assessment of Body Image Based on Large Language Model
Source: Psych J. 2025 Aug 30;14(5):669–84. doi: 10.1002/pchj.70048 (PMC12520832; doi:10.1002/pchj.70048)
Supplement: Supplementary file 1 — Data S1. Supporting Information. [file PCHJ-14-669-s001.docx]

**Supplementary Material for The Assessment of Body Image based on Large Language Model**

Prompt words for evaluating body image by large language models：

1）Perception：

>>>Prompt:

Body image refers to an individual’s mental representation of their own body, including how they perceive, evaluate, and pay attention to their appearance. The "Perception" dimension specifically reflects the individual's level of awareness, concern, and behavior related to their physical traits (e.g., weight, shape, clothing, grooming). This assessment focuses on understanding the individual’s body image perception based on their self-reflection and expressed thoughts.

Analyze the following text to evaluate the individual’s level of body image perception, focusing on the extent to which they:

Pay attention to their appearance in daily life (e.g., grooming, clothing choices, makeup).

Reflect on and evaluate specific body features (e.g., weight, shape, or other physical traits).

Show concern about others’ opinions or feedback about their appearance.

Demonstrate behaviors or habits related to maintaining or improving their physical appearance (e.g., mirror-checking, preparing for social situations).

The evaluation criteria are based on the following scale from 1.00 to 5.00 (with continuous values):

1.00 – 1.99: Very low level of body image perception. The individual shows minimal concern or awareness of their physical appearance, with few or no behaviors related to grooming, body evaluation, or concern about others’ feedback. They may rarely engage in self-reflection or behaviors to maintain or enhance their appearance.

2.00 – 2.99: Low level of body image perception. The individual shows occasional or vague awareness of their appearance but lacks consistent reflection or behavior. They may show some concern about body features or external opinions, but these behaviors are infrequent or vague.

3.00 – 3.99: Moderate level of body image perception. The individual demonstrates regular awareness of their body and appearance, with some habits (e.g., grooming, mirror-checking) and occasional concern about others’ opinions. They engage in certain behaviors related to maintaining or enhancing their appearance, but it may not be a dominant focus in their daily life.

4.00 – 4.99: High level of body image perception. The individual frequently reflects on their appearance and engages in behaviors to enhance or evaluate it (e.g., detailed grooming, choosing clothes carefully, monitoring weight). Their self-reflection and attention to physical appearance are regular and deliberate.

5.00: Very high level of body image perception. The individual shows an intense preoccupation with their appearance, engaging in frequent behaviors (e.g., makeup, weight monitoring, mirror-checking) and showing consistent concern about external feedback or maintaining their image. Their awareness of and attention to their body are prominent in daily life.

Scoring Criteria:

When scoring, consider the depth, intensity, and frequency of the following indicators:

Concern about weight or body shape (e.g., frequent mentions of weight changes, dieting, or body improvement).

Attention to grooming, clothing, and appearance preparation (e.g., time spent getting ready or evaluating outfits).

Sensitivity to others’ opinions about their appearance (e.g., external validation, fear of judgment).

Habits that reflect body image perception (e.g., frequent mirror-checking, makeup, or efforts to enhance physical appearance).

Note: This evaluation focuses on the person behind the text—inferring from their thoughts and behaviors expressed in the text. Assign a score from 1.00 to 5.00 based on the depth, intensity, and frequency of these traits. Please return only the numerical score with two decimal points without any explanation.

>>> Text to be analyzed:

2）Positive

>>> Prompt:

Body image refers to an individual’s mental representation of their own body, including perception, emotions, and behaviors. The "Positive Attitude" dimension specifically reflects the individual’s feelings of satisfaction, confidence, and self-esteem regarding their physical appearance. This assessment focuses on understanding the individual’s positive attitude toward their body image based on their self-reflection and expressed thoughts.

Analyze the following text to evaluate the individual’s level of positive body image, focusing on the extent to which they:

Express satisfaction or confidence about specific body features (e.g., face, figure, weight, height, or overall appearance).

Acknowledge and appreciate unique or attractive aspects of their physical appearance.

Demonstrate behaviors or attitudes that reflect positive emotions toward their body (e.g., self-acceptance, pride, or enjoyment of physical appearance).

Show resilience in the face of societal beauty standards, maintaining positive self-regard despite external comparisons.

Exhibit a sense of respect and care for their body (e.g., valuing its uniqueness or engaging in behaviors that demonstrate self-respect).

The evaluation criteria are based on the following scale from 1.00 to 5.00 (with continuous values):

1.00 – 1.99: Very low level of positive body image. The individual expresses no satisfaction or confidence in their appearance, possibly focusing on dissatisfaction, insecurity, or negative evaluations of their body.

2.00 – 2.99: Mild positive body image. The individual occasionally expresses satisfaction or confidence about their body but lacks a strong or consistent sense of self-appreciation. They may express vague or minimal positive emotions regarding specific body features.

3.00 – 3.99: Moderate positive body image. The individual demonstrates a general sense of satisfaction and confidence about their appearance, occasionally acknowledging positive traits or unique features. They show some level of self-acceptance and pride in their body.

4.00 – 4.99: High positive body image. The individual frequently expresses satisfaction, confidence, or pride about their appearance, with clear recognition of their unique physical traits. They actively engage in behaviors that reflect self-love, self-respect, and positive self-regard.

5.00: Very high level of positive body image. The individual consistently demonstrates a strong sense of self-esteem, pride, and appreciation for their appearance. They show resilience against societal beauty standards and regularly engage in behaviors that express deep self-respect and self-love.

Scoring Criteria:

When scoring, consider the depth, intensity, and frequency of the following indicators:

Satisfaction with specific body parts or features (e.g., "I like the way I look" or "I feel attractive").

Expressions of confidence or pride in physical appearance (e.g., "I love my figure" or "I feel good about my face").

Recognition of unique or attractive aspects of their appearance (e.g., "I appreciate my unique features" or "I love how my body is different").

Evidence of self-acceptance or positive behaviors (e.g., "I respect my body" or "I take care of my appearance because I value myself").

Resilience to societal beauty standards (e.g., "I’m not perfect, but I feel beautiful in my own way" or "I embrace my natural beauty").

Note: This evaluation focuses on the person behind the text—inferring from their thoughts and behaviors expressed in the text. Assign a score from 1.00 to 5.00 based on the depth, intensity, and frequency of these traits. Please return only the numerical score with two decimal points without any explanation.

>>> Text to be analyzed:

3）Negative

>>> Prompt:

Body image refers to an individual’s mental representation of their body, encompassing both perceptions of specific body features and the emotional and behavioral responses associated with them. The "Negative Attitude" dimension focuses on negative feelings, dissatisfaction, and self-criticism related to one’s physical appearance. This assessment aims to evaluate the extent to which the individual expresses negative emotions, insecurity, or dissatisfaction with their body image based on their self-reflection and expressed thoughts.

Analyze the following text to evaluate the individual’s level of negative body image, focusing on how much they:

Express dissatisfaction or negative emotions about specific body features (e.g., face, figure, weight, height, or overall appearance).

Acknowledge feelings of discomfort or distress due to their physical appearance, particularly in relation to how they perceive themselves compared to others.

Mention negative evaluations or criticism of their body from others (e.g., family, friends, romantic partners, or colleagues) and the emotional impact of these evaluations.

Indicate that their body image affects their emotional well-being or daily life, such as feeling upset, angry, or distressed due to their appearance.

Reflect a sense of insecurity or dissatisfaction in comparison to societal beauty standards or the appearance of others in their social environment.

The evaluation criteria are based on the following scale from 1.00 to 5.00 (with continuous values):

1.00 – 1.99: Very low level of negative body image. The individual expresses no dissatisfaction or insecurity and shows no negative emotions about their physical appearance. They may express satisfaction or acceptance of their body.

2.00 – 2.99: Mild negative body image. The individual expresses occasional or mild dissatisfaction or discomfort with their appearance. They may reference some specific body features or situations where they feel dissatisfied, but these feelings are not pervasive or deeply ingrained.

3.00 – 3.99: Moderate negative body image. The individual frequently expresses dissatisfaction or negative emotions about their physical appearance, possibly referencing specific body parts or comparing themselves to others. There may also be indications of emotional distress, insecurity, or concerns about societal standards.

4.00 – 4.99: High negative body image. The individual shows frequent and strong feelings of dissatisfaction, self-criticism, or distress about their body, often influenced by societal standards or others' negative evaluations. They may express a high level of insecurity and frustration related to their body image.

5.00: Very high negative body image. The individual exhibits intense negative emotions, such as high self-criticism, persistent dissatisfaction, or deep insecurity. They may consistently express distress caused by comparisons to others or negative evaluations from others. These feelings are overwhelming and strongly affect their emotional well-being.

Scoring Criteria:

When scoring, consider the depth, intensity, and frequency of the following indicators:

Expressions of dissatisfaction or discomfort with specific body parts (e.g., "I don’t like my face" or "I feel unattractive").

Acknowledge negative emotional reactions to body-related comments from others (e.g., "It hurts when people comment negatively on my body" or "I get upset when my partner criticizes my appearance").

Feelings of inferiority or social comparison (e.g., "I feel like I don’t measure up to others" or "I think I’m not as attractive as most people").

References to how body image issues affect daily life or emotional well-being (e.g., "I avoid social situations because of my appearance" or "I feel bad about myself when I look in the mirror").

Expressions of insecurity or frustration about body image relative to societal beauty standards (e.g., "I wish I looked like the people on TV" or "I feel like I’m not good enough").

Note: This evaluation focuses on the person behind the text—inferring from their thoughts and behaviors expressed in the text. Assign a score from 1.00 to 5.00 based on the depth, intensity, and frequency of these traits. Please return only the numerical score with two decimal points without any explanation.

>>> Text to be analyzed:

4）Behavior

>>> Prompt:

Body image refers to an individual’s mental representation of their own body, including perceptions of body parts, related emotions, and behaviors. The "Behavior" dimension specifically refers to actions taken to enhance or maintain one’s body image, such as grooming, exercising, dieting, or undergoing procedures to alter physical appearance. This assessment aims to evaluate the extent to which the individual engages in behaviors aimed at improving or altering their body image based on their self-reflection and expressed thoughts.

Analyze the following text to evaluate the individual’s level of body image-related behaviors, focusing on:

Participation in activities aimed at improving or maintaining physical appearance, such as exercise, dieting, or cosmetic procedures.

Engagement in behaviors to alter or enhance their body image (e.g., through grooming, makeup, clothing, or fitness routines).

Attempts to conceal or distract from perceived physical flaws by changing behaviors, such as altering appearance through makeup or other means.

Behavioral efforts to increase self-esteem or confidence related to body image, including actions like improving posture, enhancing personal grooming, or taking up hobbies aimed at enhancing physical attractiveness.

Behavioral efforts to alter body features, such as surgery, dieting, or fitness routines, and the frequency and intensity of these behaviors.

The evaluation criteria are based on the following scale from 1 to 5 (with continuous values):

1.00 – 1.99: Very low engagement in behaviors related to body image. The individual does not reference or engage in any noticeable behaviors aimed at improving or maintaining their physical appearance. They may show no actions or very minimal involvement in activities related to body image.

2.00 – 2.99: Low engagement in body image-related behaviors. The individual shows occasional or minimal reference to body-focused actions, such as light grooming, some exercise, or minor attention to appearance. These actions are infrequent or of low intensity.

3.00 – 3.99: Moderate engagement in body image-related behaviors. The individual engages in moderate activities related to maintaining or altering body image, such as regular exercise, diet attempts, or basic grooming habits. These behaviors are noticeable but not extreme, and may reflect some effort to enhance their physical appearance.

4.00 – 4.99: High engagement in body image-related behaviors. The individual regularly participates in significant actions aimed at improving their body image, such as exercising, dieting, undergoing cosmetic procedures, or consistently altering their appearance. Their behaviors are frequent and considerable.

5.00: Very high engagement in body image-related behaviors. The individual consistently engages in intense or extreme behaviors to enhance, alter, or maintain their body image. These actions include strict dieting, intense fitness routines, regular cosmetic surgeries, or continuous use of makeup. The individual’s behaviors are frequent, detailed, and highly focused on altering their physical appearance.

Scoring Criteria:

When scoring, consider the depth, frequency, and intensity of the behaviors mentioned in the text:

Participation in physical activities (e.g., exercise, fitness routines, sports): "I work out daily to maintain my figure" or "I regularly go to the gym."

Dieting or extreme weight management (e.g., fasting, restrictive diets, or weight loss efforts): "I follow a strict diet plan" or "I’ve tried intermittent fasting to lose weight."

Cosmetic procedures or makeup (e.g., cosmetic surgery, frequent makeup use): "I have had cosmetic surgery" or "I wear makeup every day."

Grooming and clothing choices (e.g., using clothing or makeup to alter physical appearance): "I wear specific outfits to look slimmer" or "I always dress to highlight my best features."

Behavioral efforts to conceal flaws or increase confidence (e.g., covering imperfections with makeup or altering posture): "I use makeup to cover my flaws" or "I constantly adjust my posture to appear more confident."

Note: This evaluation focuses on the person behind the text—inferring from their thoughts and behaviors expressed in the text. Assign a score from 1.00 to 5.00 based on the depth, frequency, and intensity of these behaviors. Please return only the numerical score with two decimal points without any explanation.

>>> Text to be analyzed:
